# Proteome dataset of subcutaneous adipose tissue from postpartum cows treated with sodium salicylate

**DOI:** 10.1016/j.dib.2019.104567

**Published:** 2019-09-26

**Authors:** Caio Takiya, Shawnee Montgomery, Laman Mamedova, Gitit Kra, Nataly Nemes-Navon, Yishai Levin, Sherry Fleming, Barry Bradford, Maya Zachut

**Affiliations:** a Department of Animal Sciences and Industry, Kansas State University, Manhattan 66506, United States; b Department of Ruminant Science, Institute of Animal Sciences Agricultural Research Organization/Volcani Center, Rishon Lezion 7505101, Israel; c Department of Animal Science, The Robert H. Smith Faculty of Agriculture, Food and Environment, The Hebrew University of Jerusalem, Rehovot 76100, Israel; d The Nancy and Stephen Grand Israel National Center for Personalized Medicine, Weizmann Institute of Science, Rehovot 7610001, Israel; e Division of Biology, Kansas State University, Manhattan 66506, United States

**Keywords:** Adipose tissue, Inflammation, Non-steroidal anti-inflammatory drug, Proteomics, Transition period

## Abstract

This article contains raw and processed data related to research published by Takiya et al. [1]. Literature have shown that treatment with the nonsteroidal anti-inflammatory agent sodium salicylate (**SS**) during the first days postpartum in dairy cows increased lactation performance [2], and tended to alter glucose metabolism [3]. Yet, the specific effects of systemic SS treatment on the proteome of the adipose tissue (**AT**) and on the inflammatory process in AT of postpartum cows is unknown. Subcutaneous AT samples were collected at 7 d of lactation from control cows (n = 5) and from cows treated with 2.3 g/L SS (n = 5) via drinking water during the first 7 d of lactation. Protein extraction and liquid chromatography-mass spectrometry were performed to obtain proteomics data. Differential abundance of proteins was determined through MS1 intensity based label-free method. Proteomics analysis generated a novel dataset consisted of 1422 proteins, 80 (5.6%) of which were differentially abundant [fold change ± 1.5, *P* < 0.05 2-way ANOVA] when comparing control and SS-treated cows. The present dataset of subcutaneous AT proteome from postpartum dairy cows treated with SS can be used as a reference for any research involving nonsteroidal anti-inflammatory agent treatment in dairy cows or in comparative research between species.

**Table:** Specifications Table

Subject	Biology
Specific subject area	Physiology
Type of data	Table
How data were acquired	Liquid Chromatography-Mass spectrometry: nanoAcquity + Q Exactive Plus
Data format	Raw Identifications Analyzed Filtered
Parameters for data collection	Protein abundance in subcutaneous adipose tissue from the tail-head region of 10 dairy cows
Description of data collection	Subcutaneous adipose tissue was sampled from the tail-head region of 5 control dairy cows and 5 that were treated daily with sodium salicylate (2.3 g/L) via drinking water. Adipose tissue was analyzed through Liquid Chromatography-Mass spectrometry following protein extraction. Differential abundance was quantified using MS1 intensity based label-free.
Data source location	Institution: Kansas State University City/Town/Region: Manhattan, Kansas Country: United States Latitude 39.183609, longitude −96.571671, 39° 11′ 0.9924″ N, and 96° 34′ 18.0156″ W
Data accessibility	With the article
Related research article	Author's name: Caio Seiti Takiya Title: Proteomic analysis reveals greater abundance of complement and inflammatory proteins in subcutaneous adipose tissue from postpartum cows treated with sodium salicylate Journal: Journal of Proteomics https://doi.org/10.1016/j.jprot.2019.103399

**Table:** 

**Value of the Data** • This is the first documentation of proteomic dataset from subcutaneous adipose tissue (AT) in postpartum dairy cows that were treated with SS, quantifying 1422 proteins. • The proteome dataset from subcutaneous AT can be used as a reference for any research involving the use of nonsteroidal anti-inflammatory treatment to postpartum dairy cows. • This data can be utilized in pharmacological research interested in the global effects of SS treatment on peripheral tissues in mammals. • The dataset of AT proteome may be useful in comparative research between species.

## 1 Data

This data describes the proteome of subcutaneous adipose tissue (AT) from postpartum dairy cows that were either controls or treated with 2.3 g/L of SS in drinking water until 7 d in lactation. The proteomic analysis was conducted following previous work that showed effects of SS treatment in lactating cows [[2], [3]]. Supplementary Table 1 contains the dataset of 1422 identified and quantified proteins obtained by proteomic analysis, as well as statistical analysis of differentially abundant proteins between SS and control cows.

## 2 Experimental design, materials, and methods

### 2.1 Animals and procedures

The experiment was conducted after the approval by the Kansas State University Institutional Animal Care and Use Committee (protocol 3182). Detailed animal management and handling can be found in Takiya et al. [1]. Ten multiparous Holstein dairy cows were assigned to either control (CON) or sodium salicylate (SS, 2.3 g/L in drinking water; Wintersun Chemical, Ontario, CA) treatment after parturition (4–36 h). Treatments were provided during the first 7 d of lactation and subcutaneous AT from the tail-head region was sampled on day 7 for proteomic analysis. Details on cows, procedures, and samplings are described in Ref. [1].

### 2.2 Sample processing, liquid chromatography, and mass spectrometry

Adipose tissue sample preparation and proteomic analysis were carried out as described in Zachut [4]. In short, AT protein concentration was determined using bicinchoninic acid assay. Samples were lysed using SDT lysis buffer, centrifuged, and supernatant (50 μg) collected. Supernatant was then mixed with 200 mL of urea buffer, filtered (30-kDa cutoff filtered), centrifuged, and washed with urea buffer. Iodoacetamide was added to the previous solution, incubated for 10 min, centrifuged, and washed twice with ammonium bicarbonate. Samples were then submitted to tryptic digestion (1 μg trypsin +40 μL of ammonium bicarbonate) overnight at 37 °C. After digestion, proteins were acidified (trifluoroacetic acid), and desalted in a solid-phase extraction column (Oasis HLB, Waters, Milford, MS, USA). Split-less nano ultra-performance liquid chromatography (UPLC; 10K psi nanoAcquity, Waters) was performed using the following mobile phases: (A) H_2_O + 0.1% (v/v) formic acid and (B) acetonitrile + 0.1% formic acid. Online samples desalting was performed in a reverse-phase C18 trapping column. Separation of peptides was made using an HSS T3 nano-column. Peptide elution into the mass spectrometer used the gradients described in Takiya et al. [1]. Ultra LC–MS-grade solvents were used for all chromatographic steps (Bio-Lab, Jerusalem, Israel). Mass spectrometer (Q Exactive Plus, Thermo Scientific, Watham, MA, USA) coupled online with nano-UPLC through a nano-ESI emitter (10-μm tip; New Objective, Woburn, MA, USA) was used to identify proteins. Data were retrieved in data-dependent acquisition mode using a Top20 method. Settings on quadrupole isolation window, MS1 and MS2 resolutions, and normalized settings and exclusion criteria are described in Takiya et al. [1].

### 2.3 Data processing

An intensity-based label-free proteomics method was used to quantify proteins [5] using the Expressionist software (version 9.2.4; Genedata, Basal, Switzerland). Detailed data processing and analysis is described in Takiya et al. [1]. Briefly, detection was dependent on peak volume in retention time, isotopic clustering, and *m*/*z* and intensity space. Peak list generated from MS/MS events was compared with the Mascot v2.5.1 (Matrix Sciences, Boston, MA, USA) database against *Bos taurus* sequences in UniprotKB (http://www.uniprot.org/). Fixed and variable modifications were set to carbamidomethylation of cysteines and oxidation of methionines, respectively. Results of search were imported to Scaffold (version 3.5, Proteome Software, Portland, OR) for filtering. A maximum of 1% false discovery rate was set by the embedded ProteinProphet algorithm. Grouping and quantification of proteins were carried out using an in-house script [5]. Proteins were quantified according to the three most abundant peptides within protein, except when only 2 or less peptides were detected. Data was normalized based on the ion current. Abundance of proteins was determined using the iBAQ method. Data global integrity and outliers were analyzed through principal component analysis.

### 2.4 Statistical analysis

Two clusters were identified by principal component analysis of the proteomic data; however, cluster was not related with treatment (*P* = 0.99). On the other hand, cluster was predictive of protein concentration of the initial subcutaneous AT lysate (*P* < 0.01); thus, the cluster effect was considered in the statistical model. Proteomics data were log transformed and analyzed by 2-way ANOVA (Statmodel of Python, version 3.6.4) to evaluate the effects of cluster, treatment, and the interaction between treatment and cluster. Abundance of proteins for each effect was considered different when *P* < 0.05 and an absolute fold change (FC) ± 1.5. The data analysis was based on the strategy of Feise [6]. Fold changes were calculated as the ratio of arithmetic means of the sodium salicylate versus control samples.
